# Screening of Biomarkers and Toxicity Mechanisms of Rifampicin-Induced Liver Injury Based on Targeted Bile Acid Metabolomics

**DOI:** 10.3389/fphar.2022.925509

**Published:** 2022-06-10

**Authors:** Yang Deng, Xilin Luo, Xin Li, Yisha Xiao, Bing Xu, Huan Tong

**Affiliations:** ^1^ Department of Pharmacy, The Third Hospital of Changsha, Changsha, China; ^2^ The Clinical Application Research Institute of Antibiotics in Changsha, Changsha, China

**Keywords:** rifampicin, liver injury, targeted bile acid metabolomics, farnesoid x receptor, multidrug resistance-associated proteins

## Abstract

Rifampicin (RIF) is a critical first-line drug for tuberculosis. However, long-term or high-dose treatment with RIF can induce severe liver injury; the underlying mechanism of this effect has not yet been clarified. This study was performed to screen reliable and sensitive biomarkers in serum bile acids (BAs) using targeted BA metabolomics and evaluate the toxicity mechanisms underlying RIF-induced liver injury through the farnesoid x receptor (Fxr)-multidrug resistance-associated proteins (Mrps) signaling pathway. Thirty-two Institute of Cancer Research mice were randomly divided into four groups, and normal saline, isoniazid 75 mg/kg + RIF 177 mg/kg (RIF-L), RIF-L, or RIF 442.5 mg/kg (RIF-H) was orally administered by gavage for 21 days. After treatment, changes in serum biochemical parameters, hepatic pathological conditions, BA levels, Fxr expression, and BA transporter levels were measured. RIF caused notable liver injury and increased serum cholic acid (CA) levels. Decline in the serum secondary BAs (deoxycholic acid, lithocholic acid, taurodeoxycholic acid, and tauroursodeoxycholic acid) levels led to liver injury in mice. Serum BAs were subjected to metabolomic assessment using partial least squares discriminant and receiver operating characteristic curve analyses. CA, DCA, LCA, TDCA, and TUDCA are potential biomarkers for early detection of RIF-induced liver injury. Furthermore, RIF-H reduced hepatic BA levels and elevated serum BA levels by suppressing the expression of Fxr and Mrp2 messenger ribonucleic acid (mRNA) while inducing that of Mrp3 and Mrp4 mRNAs. These findings provide evidence for screening additional biomarkers based on targeted BA metabolomics and provide further insights into the pathogenesis of RIF-induced liver injury.

## 1 Introduction

Tuberculosis (TB) is an infectious disease caused by *Mycobacterium tuberculosis*, the second leading cause of death from a single infectious agent after severe acute respiratory syndrome coronavirus 2 (World Health Organization, 2021). Treatment for drug-susceptible TB requires a combination of rifampicin (RIF), isoniazid (INH), ethambutol, and pyrazinamide for 2-months followed by RIF and INH for 4 months, while 6–20 months for multidrug-resistant TB (Lange et al., 2019; World Health Organization, 2021). Prolonged RIF administration or the combination of RIF and INH would significantly increase the risk of drug-induced liver injury with an undefined mechanism (Devarbhavi et al., 2021). The current standard RIF dose of 10 mg/kg daily is suboptimal and has remained unchanged since the early 1970s (Grobbelaar et al., 2019; Te Brake et al., 2021). In RIF clinical trials, doses of 20, 25, 30, 35, and 50 mg/kg have been administered. The safety of RIF 50 mg/kg should be verified in further investigation, given its poor tolerance in humans (Boeree et al., 2015; Abulfathi et al., 2019; Te Brake et al., 2021). Long-term RIF treatment can cause hepatocyte dysfunction and cholestatic liver injury (Grobbelaar et al., 2019; Yang et al., 2020c). The mechanism of RIF-induced drug-induced liver injury (DILI) remains undefined and may occur by cholestasis, oxidative stress, inflammatory response, mitochondrial damage, and apoptosis (Moussavian et al., 2016; Yang et al., 2020c; Luo et al., 2021).

Bile acids (BAs), a major component of bile, regulate glucose and lipid metabolism, as well as energy homeostasis (Chiang and Ferrell, 2020). A previous study indicated that the disorder of BAs was associated with primary sclerosing cholangitis, primary biliary cholangitis, previously known as primary biliary cirrhosis, nonalcoholic fatty liver, and nonalcoholic steatohepatitis (Choudhuri and Klaassen, 2022). It is essential to evaluate therapeutic agents to treat liver injury and elucidate the relationship between BA metabolism and the mechanisms of liver injury. Farnesoid x receptor (Fxr), which is abundantly expressed in the liver, gastrointestinal tract, kidney, and adrenal glands, plays an essential role in regulating BA and lipid metabolism and glucose homeostasis. Fxr is activated by chenodeoxycholic acid (CDCA), cholic acid (CA), deoxycholic acid (DCA), lithocholic acid (LCA), and their taurine and glycine conjugates, and promotes BAs flow (Yang et al., 2017; Gottlieb and Canbay, 2019; Keitel et al., 2019; Chiang and Ferrell, 2020). Fxr represses the *de novo* synthesis and uptake of BAs in the liver and protects hepatocytes from the accumulation of toxic BAs under cholestatic conditions. Multidrug resistance-associated proteins (Mrps) are BAs efflux transporters, the regulation of BAs by Fxr is related to the regulation of subtypes of Mrps, such as Mrp2, Mrp3, and Mrp4 (Xu et al., 2005; Yuan et al., 2018; Fiorucci et al., 2021). Uridine diphosphate-glucuronosyltransferases (UGTs), which can co-express Mrp2, are associated with the phase II metabolic pathway. UGTs substrates are metabolized by glucuronidation, increasing hydrophilicity and facilitating the excretion of conjugated metabolites into bile and urine, thus further reducing toxicity (Xu et al., 2005). Furthermore, the prognosis of DILI can be improved by considering and detecting changes in BA efflux transporter levels (Ali et al., 2017).

Serum alanine aminotransferase (ALT) and aspartate aminotransferase (AST) levels are less sensitive and liver-specific for DILI relative to the new biomarkers, such as BAs and glutamate dehydrogenase (McGill and Jaeschke, 2019; Tian et al., 2022). The reason for the increase in ALT activity is also correlated with skeletal muscle, cardiac injury or metabolic state, except for histopathology change (Zhao et al., 2017). Moreover, a previous study found that the levels of hepatic and serum BAs and BA-related genes increase notably, with no elevation in serum ALT, AST, and alkaline phosphatase levels after administering a dose of RIF (Sanoh et al., 2019). Another study showed that the levels of serum conjugated BAs [glycocholic acid (GCA) and taurocholic acid (TCA)] increase in the rats with bile duct hyperplasia without significant changes in ALT and AST levels (Slopianka et al., 2017). It was shown that ALT and AST indicated lower sensitivity and specificity for the detection of liver injury than BAs. BA metabolism was closely related to the pathologic changes in the hepatic and biliopancreatic diseases. Since the 1950s, enzymatic methods of quantifying the “total” bile acid pool in blood have been established (Hurlock and Talalay, 1957). In recent years, many metabolomics studies have demonstrated that BAs are potential biomarkers for the diagnosis, follow-up and prognosis of liver injury and dysfunction (Ambros-Rudolph et al., 2007; Crosignani et al., 2007; Dong et al., 2015; Tian et al., 2022). Tian and others found that serum glycoursodeoxycholic acid (GUDCA), taurodeoxycholic acid (TDCA), and taurolithocholic acid (TLCA) were helpful for evaluating Cd-induced liver injury and Cd exposure in humans based on targeted BA metabolomics (Tian et al., 2022). In another study, glycodeoxycholic acid (GDCA) in the bile and hyodeoxycholic acid (HDCA) in the serum could be potential biomarkers for *Polygonum multiflorum*-induced liver injury (Dong et al., 2015). Therefore, analysis of individual BAs has the potential to unravel valuable biomarkers for the differentiation and diagnosis of various forms of liver injury (Luo et al., 2018). It is necessary to adopt targeted BA metabolomic approaches using liquid chromatography-tandem mass spectrometry (LC-MS/MS) to identify potential biomarkers from serum BAs.

In a study of liver injury in mice, RIF treatment suppressed Na^+^/taurocholate cotransporter, one of the targets of Fxr signaling (Yang et al., 2020b). Besides, it is clear that the expression levels of organic solute transporter *β* were increased following treatment with RIF (Zhang et al., 2017). Mrps play an essential role in disorders of BAs metabolism and DILI (Cuperus et al., 2014). The relationship between the change of Fxr and Mrp2, Mrp3, Mrp4 mRNA, and RIF-induced DILI is still insufficient. Therefore, Our study aims to establish an experimental mouse model of RIF-induced liver injury to analyze the changes in the metabolic profile of BAs in the liver and serum using LC-MS/MS and unravel the potential serum biomarkers for liver injury. Comprehensive analysis of the role of the Fxr-Mrps signaling pathway in BA metabolism, we could better understand the hepatotoxicity mechanism of RIF action and further provide a theoretical basis for DILI prevention and rational clinical application in anti-TB therapy.

## 2 Materials and Methods

### 2.1 Chemicals and Reagents

RIF and INH were purchased from Meilun Biotechnology Co. Ltd (Dalian, China). Ursodeoxycholic acid (UDCA), HDCA, CA, CDCA, DCA, tauroursodeoxycholic acid (TUDCA), and taurochenodeoxycholic acid (TCDCA) were purchased from On-Road Biotechnology Co., Ltd (Changsha, China). TCA, LCA, GCA, glycochenodeoxycholic acid (GCDCA), GDCA, TLCA, and TDCA were purchased from Sigma Reagents, Inc (St. Louis, MO, United States). Taurohyodeoxycholic acid (THDCA) and GUDCA were purchased from Aikeda Chemical Reagent Co., Ltd (Chengdu, China). Cyproterone acetate was purchased from China National Institutes for Food and Drug Control (Beijing, China). Acetonitrile and methanol (chromatographic grade purity) were purchased from Merck (Darmstadt, Germany). Formic acid and ammonium acetate (chromatographic grade purity) were purchased from ROE Scientific Inc (United States). The EVOM-MLV reverse transcription kit, SYBR Green Premix Pro Taq HS premixed quantitative real-time polymerase chain reaction (PCR) kit, nuclease-free water, and AG RNAex Pro ribonucleic acid (RNA) extraction reagent were purchased from Aicerui Biological Engineering Co., Ltd. (Hunan, China).

### 2.2 Animals and Experimental Design

**FIGURE 1:**
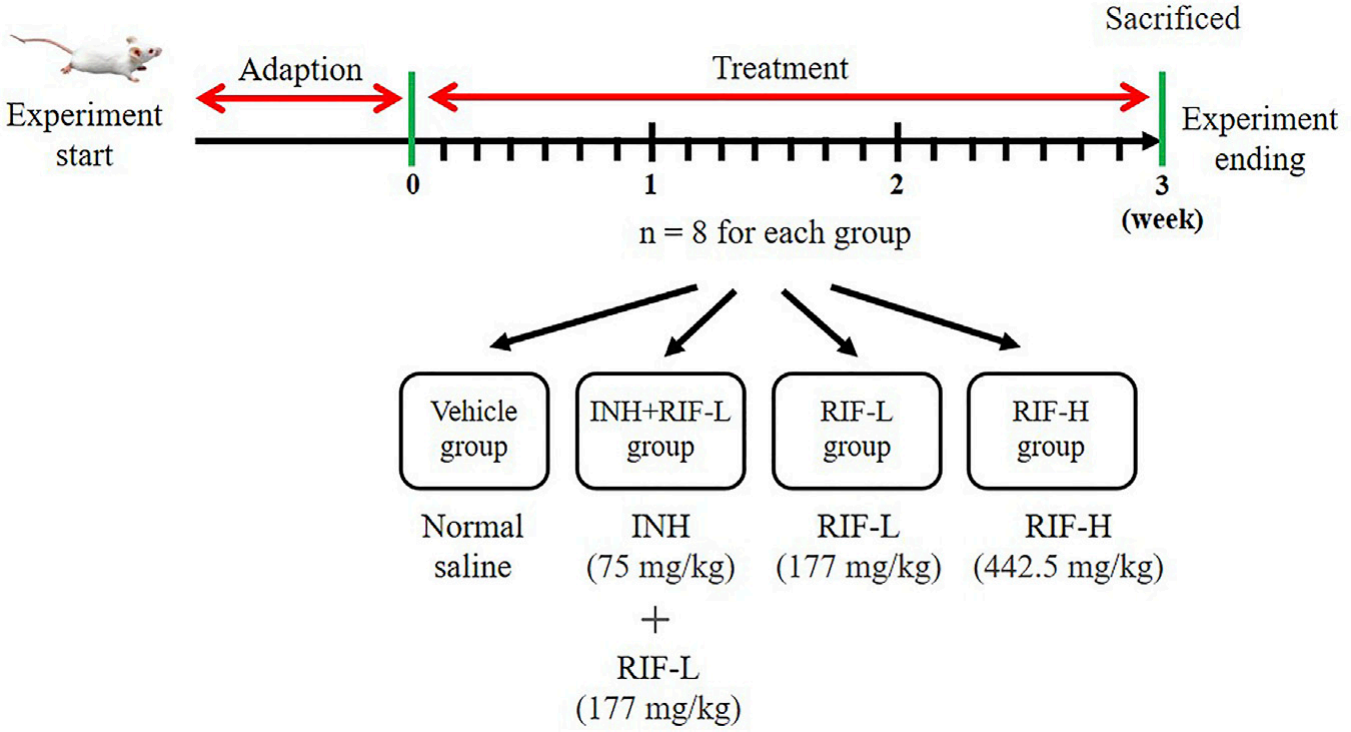
The scheme of animal experiment. The mice were divided into four groups: vehicle, INH (75 mg/kg)+RIF-L (144 mg/kg), RIF-L, and RIF-H (442.5 mg/kg) group, with eight mice in each group.

All animal experiments were conducted in compliance with the Guide for the Care and Use of Laboratory Animals. Healthy Institute of Cancer Research mice, weighing 18–22 g, were provided by Hunan Slack Jingda Experimental Animal Co., Ltd (Hunan, China). The mice were provided *ad libitum* access to water and chow and kept at 20 ± 2°C at 50 ± 10% relative humidity with a 12-h/12-h light/dark cycle. Thirty-two mice were randomly divided into four groups (eight mice in each group) and treated as follows: normal saline (vehicle), INH 75 mg/kg + RIF 177 mg/kg (RIF-L), RIF-L, and RIF 442.5 mg/kg (RIF-H). RIF and INH were administered daily by gavage for 21 days (Figure 1). After day 21, the mice fasted for 12 h before anatomical examination. Blood samples and liver tissues from the mice were collected for further experiments. The animal experiment was approved by Animal Ethics Review Regulations of Hunan Academy of Traditional Chinese Medicine and was reviewed and approved by the Animal Ethics Review Committee. (Number:2019-0024).

### 2.3 Biochemical and Histological Analyses

ALT, AST, total bilirubin (TBil), and indirect bilirubin (IBil) levels and liver index (liver weight/body weight ×100) were measured as described earlier (Luo et al., 2020). The fixed liver tissue was embedded in paraffin blocks. Next, 4-μm-thick slices were cut and stained with hematoxylin and eosin to investigate histopathological changes.

### 2.4 LC-MS/MS for Liver and Serum BAs

#### 2.4.1 Instruments and Conditions

LC-MS/MS for BAs (Supplementary Figure S1) was performed using the LCMS-8050 triple quadrupole mass spectrometer (Shimadzu, Japan). The separation was performed on an Ultimate AQ-C18 (3.0 mm × 100 mm, 3.0 μm; Welch, United States) analytical column connected with a top C18 column (Guard cartridge System, United States). The column temperature was maintained at 40°C. The gradient system consisted of solvent A (0.005% formic acid containing 7 mmol/L ammonium acetate) and solvent B (methanol) at a flow rate of 0.60 ml/min, and the gradient program was as follows: 40%:60% (v/v, 0–2 min), 5%:95% (v/v, 13.0–17.3 min), and 40%:60% (v/v, 17.4–29.3 min). Cyproterone acetate was used as the internal standard (IS). The multiple reaction monitoring functions used MS in the electrospray ionization negative mode. Other MS parameters were as follows: Interface: ESI(-); Drying gas flow: 10.0 L/min; Heat block temperature: 400°C. The multiple reactions monitoring the ion pairs of the BAs were listed in Table 1.

**TABLE 1:** The optimum LC-MS/MS working parameters for BAs.

BAs	MRM m/z	CE/V-	Dwell time (msec)	Retention time (min)
CA	407.30/407.30	10.0	50.0	11.83
CDCA	391.30/391.30	10.0	50.0	14.27
DCA	391.30/391.30	10.0	50.0	14.70
LCA	375.50/375.50	10.0	50.0	16.45
UDCA	391.30/391.30	10.0	50.0	9.80
HDCA	391.30/391.30	10.0	50.0	10.80
TCA	514.20/80.00	55.0	50.0	10.40
TCDCA	498.20/80.05	55.0	50.0	12.80
TDCA	498.20/80.05	55.0	50.0	13.39
TLCA	482.20/80.00	55.0	50.0	14.98
TUDCA	498.20/80.05	55.0	50.0	8.06
THDCA	498.20/80.05	55.0	50.0	9.04
GCA	464.20/74.00	38.0	50.0	10.62
GCDCA	448.20/73.90	37.0	50.0	13.03
GDCA	448.20/73.90	37.0	50.0	13.60
GUDCA	448.20/73.90	37.0	50.0	8.20

#### 2.4.2 Sample Preparation

To analyze 16 BAs in the mouse liver, liver tissue was weighed and added to the saline solution according to the ratio of 1:10, and the mixtures were homogenized using a tissue homogenizer (Servicebio, China). After the homogenation, 50 µL hepatic homogenate was added to 150 µL of IS solution. The mixture was vortexed for 2 min before centrifugation at 4°C and 10,000 g for 10 min. The supernatant (150 µL) was volatilized to dryness in a centrifugal vacuum concentration system, carefully collected, and resuspended in 50 μL 50% methanol. Finally, the supernatant was injected into the LC-MS/MS system. The IS method was used for quantitation. For each serum sample, the experiment was processed separately as described above. That is, 50 µL serum was added to 150 µL of the IS solution, and the other processes remained unchanged.

### 2.5 Quantitative Real-Time PCR

Quantitative real-time PCR was performed on the ABI 7500 Fast Real-Time PCR system (Thermo Fisher Scientific, United States). Total RNA was extracted using the reverse transcription kit with the TRIzol reagent and reverse-transcribed into complementary deoxyribonucleic acid (cDNA). cDNA was amplified and analyzed using the SYBR Green Premix Pro Taq HS qPCR kit (Ackeri Bioengineering Co., Ltd.). Glyceraldehyde-3-phosphate dehydrogenase (*Gapdh*) was used as a control. The primer sequences used in our study are listed in Table 2.

**TABLE 2:** Primer sequences used for quantitative real-time PCR.

Gene	Primer
glyceraldehyde-3-phosphate dehydrogenase	Forward: 5′-AGG​TCG​GTG​TGA​ACG​GAT​TTG-3′
	Reverse: 5′-GGG​GTC​GTT​GAT​GGC​AAC​A-3′
Fxr	Forward: 5′-AGC​TAA​TGA​GGA​CGA​CAG​CG-3′
	Reverse: 5′-TGC​CGT​GAG​TTC​CGT​TTT​CT-3′
Mrp2	Forward: 5′-TGA​GGA​AGA​GGA​TGG​TGA​CTG​TGG-3′
	Reverse: 5′-GTT​CGG​CGA​AGG​CTG​TTC​TCC-3′
Mrp3	Forward: 5′-ATC​ACC​ATA​CAC​AAC​GGC​ACC​TTC-3′
	Reverse: 5′-TCC​GAG​TAA​GGC​AGA​CAC​CAG​AG-3′
Mrp4	Forward: 5′-TCT​ACC​AGG​ACG​CCG​ACA​TCT​AC-3′
	Reverse: 5′-ACA​GTT​GGA​ACA​GGT​GCT​TGC​C-3′
UGT1a1	Forward: 5′-TTC​CTG​TGC​CTC​TCC​TTT​AAC​T-3′
	Reverse: 5′-TCA​TCC​AGT​CAA​ACC​AGC​C-3′

### 2.6 Statistical Analyses

Partial least squares-discriminant analysis (PLS-DA) is accomplished by linking two data matrices X and Y to maximize the covariance between the independent variables X and the corresponding dependent variable Y of highly multidimensional data by finding a linear subspace of the explanatory variables. VIP (variable importance) is mainly used for screening the important variables. The technology of VIP can be used in the case of small sample size and a strong correlation between several independent variables. It means an important variable in the model with a value of VIP ≥1 (Gromski et al., 2015; Lee et al., 2018). Differential markers could be screened by VIP value and SPSS statistical analysis of PLS-DA variables (*p* < 0.05). PLS-DA is widely used for the data analysis of metabolomics. Simca-p 14.1 software was used for PLS-DA to assess the data. VIP was calculated for the liver and serum BA levels using this software. Statistical and receiver operating characteristic (ROC) curve analyses were performed using GraphPad Prism eight and SPSS 25.0, respectively, following recommendations in the pharmacology field. All experimental data obtained in this study are presented as the mean ± standard deviation. Data from multiple groups were compared using a one-way analysis of variance, followed by the least significant difference test. Correlation analysis was performed using Pearson’s test. Differences were considered statistically significant at *p* < 0.05.

## 3 Results

### 3.1 Effects of RIF on Biochemical and Histological Changes

**FIGURE 2:**
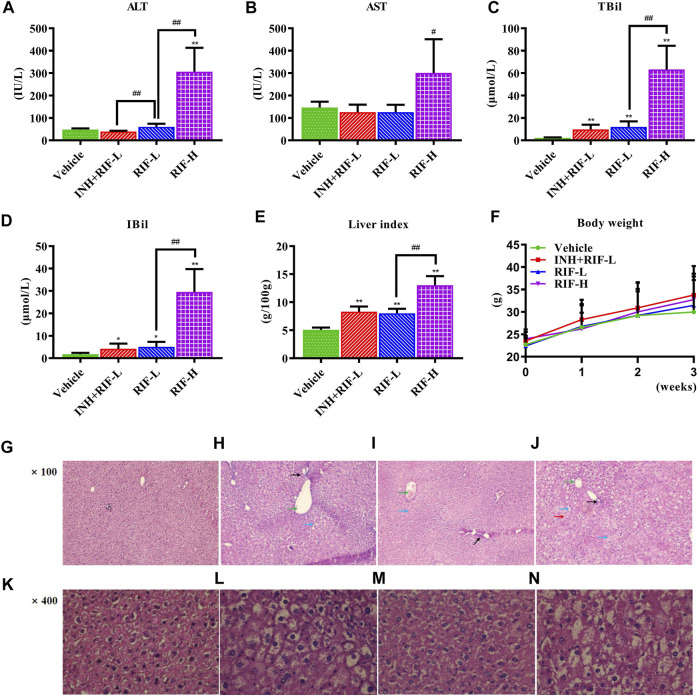
Effects of RIF on serum biomarkers, liver indexes, and histological parameters (HE staining) **(A)** ALT **(B)** AST **(C)** TBil **(D)** IBil **(E)** Liver index **(F)** Body weight **(G)** The vehicle group in HE, ×100 **(H)** The INH + RIF-L group in HE, ×100 **(I)** The RIF-L group in HE, ×100 **(J)** The RIF-H group in HE, ×100 **(K)** The vehicle group in HE, ×400 **(L)** The INH + RIF-L group in HE, ×400 **(M)** The RIF-L group in HE, ×100 **(N)** The RIF-H group in HE, ×400. Data are expressed as mean ± SD (*n* = 6). *p < 0.05, **p < 0.01 versus the vehicle group; #*p* < 0.05, ##*p* < 0.01 versus the RFP-L group.

To evaluate RIF-induced liver injury, some biochemical indicators were analyzed. As exhibited in Figure 2, compared with the vehicle group, the serum levels of ALT, AST, TBil, IBil, and liver index in the RIF-H group were increased by 6.4-, 2.1-, 29.1-, 17.0-, and 2.6- fold, respectively; but only TBil, IBil, and the liver index increased significantly in the INH + RIF-L and RIF-L groups. Histopathological analysis showed that a typical structure with well-arranged hepatocyte cords and obvious hepatic sinusoids was observed in the vehicle group. The liver sections of mice treated with INH + RIF-L and RIF-L showed a typical arrangement of the hepatic cords and central veins, except for some nuclear disappearance and partial vacuoles. Meanwhile, biliary duct dilatation features can be observed in the INH + RIF-L and RIF-L groups, marked in Figures 2H,I, respectively. The RIF-H group showed steatosis, hepatocyte swelling, dissolution, the disappearance of the nucleus, and increased intracellular damage (Luo et al., 2020). These results indicated that RIF induced hepatoxicity in a dose-dependent manner **(** Figure 2 **)**.

### 3.2 Changes in Liver and Serum BA Profiles

**FIGURE 3:**
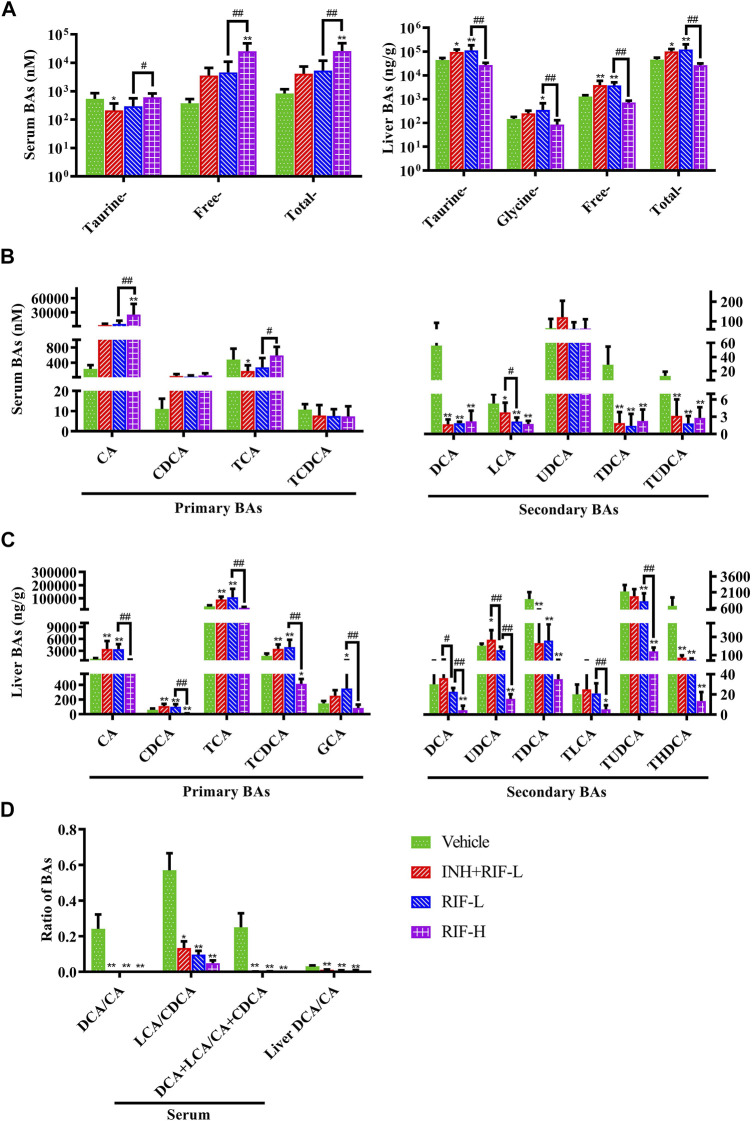
Effects of RIF on the serum and liver BA levels in mice **(A)** Taurine, free, total BAs in the serum and liver **(B)** Primary and secondary BAs in the serum **(C)** Primary and secondary BAs in the liver **(D)** The ratio of secondary BAs to primary BAs. Data are expressed as mean ± SD (*n* = 5–8). *p < 0.05, **p < 0.01 versus the vehicle group; #*p* < 0.05, ##*p* < 0.01 versus the RFP-L group. Green arrows indicate dilatation of central veins, blue arrows indicate hepatocellular hydropic degeneration, red arrow indicate necrosis, black arrows indicate cholangiectasis.

LC-MS/MS was performed to detect the liver and serum BA profiles **(** Figure 3 **)**. The results showed that the levels of serum-free and total BAs were significantly elevated in the RIF-H group. Serum CA was upregulated in the RIF-H group, whereas secondary BAs (DCA, LCA, TDCA, and TUDCA) were downregulated in all the RIF-administered mice.

The levels of hepatic taurine-conjugated, free, and total BAs significantly increased in the RIF-L and INH + RIF-L groups. In addition, RIF significantly increased the levels of hepatic primary BAs such as CA, CDCA, TCA, and TCDCA in the RIF-L and INH + RIF-L groups. However, the levels of all hepatic BAs reduced considerably in the RIF-H group.

RIF, administered alone or in combination with INH, significantly reduced the ratio of secondary to primary BAs in the liver and serum (*p* < 0.01 or 0.05), including DCA/CA, LCA/CDCA, and LCA + DCA/CDCA + CA **(** Figure 3 **)**.

### 3.3 Screening Targeted BAs for RIF-Induced Liver Injury in Mice

**FIGURE 4:**
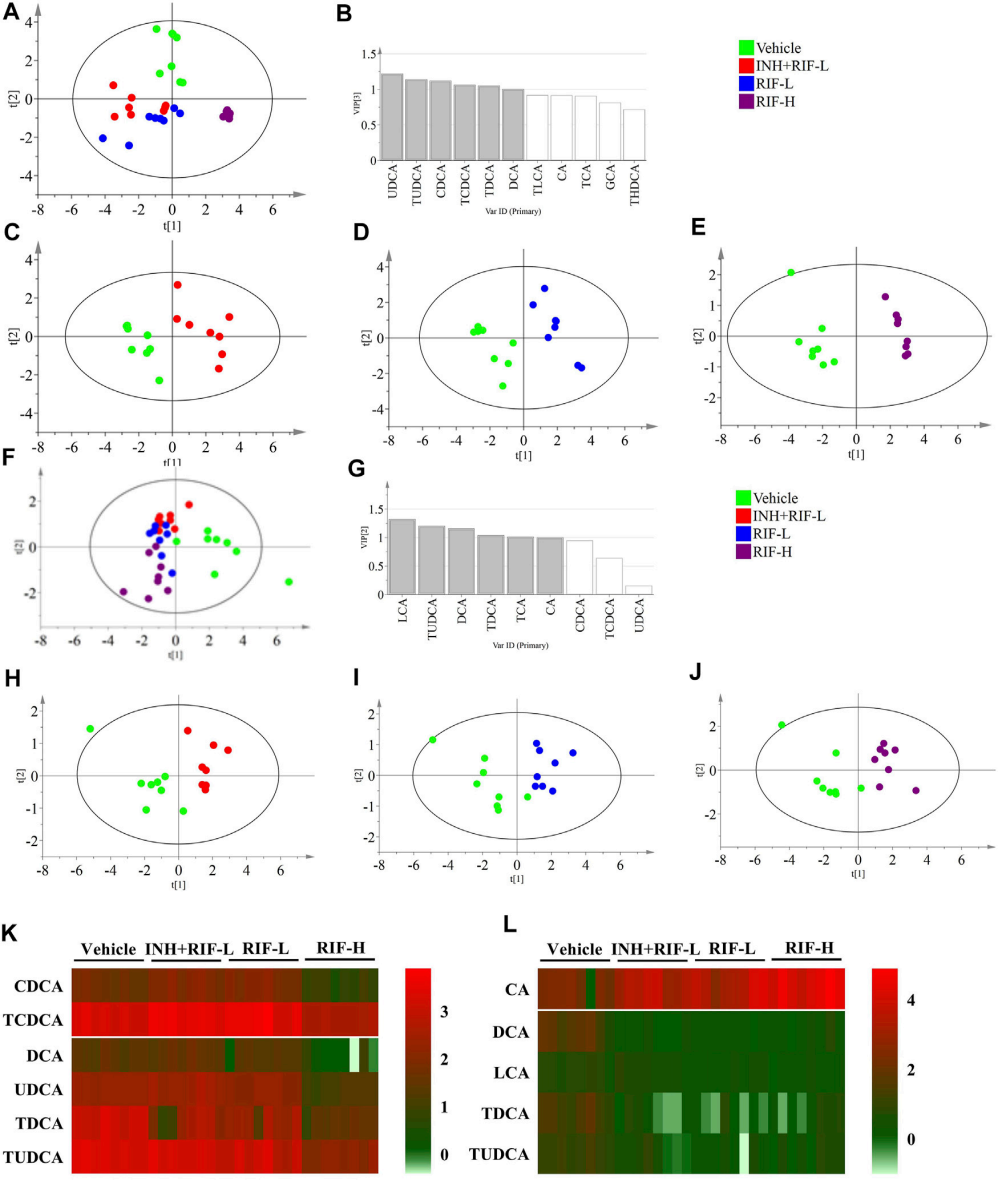
Targeted metabolomics of BA profiles **(A)** Score plots of PLS-DA for the liver in the vehicle, INH + RIF-L, RIF-L, and RIF-H groups **(B)** VIP value in the liver between the vehicle and RIF-H groups **(C)** Score plots of PLS-DA of the liver between the vehicle and INH + RIF-L groups **(D)** Score plots of PLS-DA of the liver between the vehicle and RIF-L groups **(E)** Score plots of PLS-DA of the liver between the vehicle and RIF-H groups **(F)** Score plots of PLS-DA of the serum among the vehicle, INH + RIF-L, RIF-L, and RIF-H groups **(G)** VIP value for the serum between the vehicle and RIF-H groups **(H)** Score plots of PLS-DA of the serum between the vehicle and INH + RIF-L groups **(I)** Score plots of PLS-DA of the serum between the vehicle and RIF-L groups **(J)** Score plots of PLS-DA of the serum between the vehicle and RIF-H groups **(K)** Heatmap of BAs (VIP ≥1) in the liver **(L)** Heatmap of serum potential BAs biomarkers. Data are expressed as mean ± SD (*n* = 5–8).

Targeted metabolomic analysis was performed to examine the changes in BA homeostasis using PLS-DA **(** Figure 4 **)**. The analysis revealed a distribution pattern in the four groups of mice. Scatter plots were clearly separated for the four groups in the liver (cumulative R2X = 80.2%, cumulative R2Y = 63.7%, cumulative Q2Y = 55.7%) and the serum BAs (cumulative R2X = 57.1%, cumulative R2Y = 45.2%, cumulative Q2Y = 37.3%). Targeted metabolomic analysis of BAs was performed between the vehicle and other groups. The explanatory ability (R2X) of liver modeling consisting of the vehicle and INH + RIF-L groups, the vehicle and RIF-L groups, and the vehicle and RIF-H groups were 74.3, 66.5, and 89.1%, respectively; the stability of modeling (R2Y) was 94.2, 94.5, and 99.4%, respectively; the predictability of modeling (Q2Y) was 80.8, 90.8, and 97.5%, respectively. In parallel, in the serum, R2X of modeling was 59.3, 62.2, and 61.1%; R2Y was 82.3, 80.2, and 86.9%; and Q2Y was 40, 54.1, and 73.2%, respectively.

BAs with VIP ≥1 were UDCA, TUDCA, CDCA, TCDCA, TDCA, and DCA in the liver in the PLS-DA modeling consisting of the vehicle and RIF-H groups, and LCA, TUDCA, DCA, TDCA, and CA in the parallel serum modeling. The variable BAs with VIP ≥1 were modeled for re-analysis, and the R2X of the liver BA model between the vehicle and RIF-H groups improved to 92.4%. The serum R2X level of the corresponding model was 82.3%. After re-analysis, the screening variables in models with VIP ≥1 showed notable improvement in the explanatory ability, maintenance of high stability, and predictability.

**FIGURE 5:**
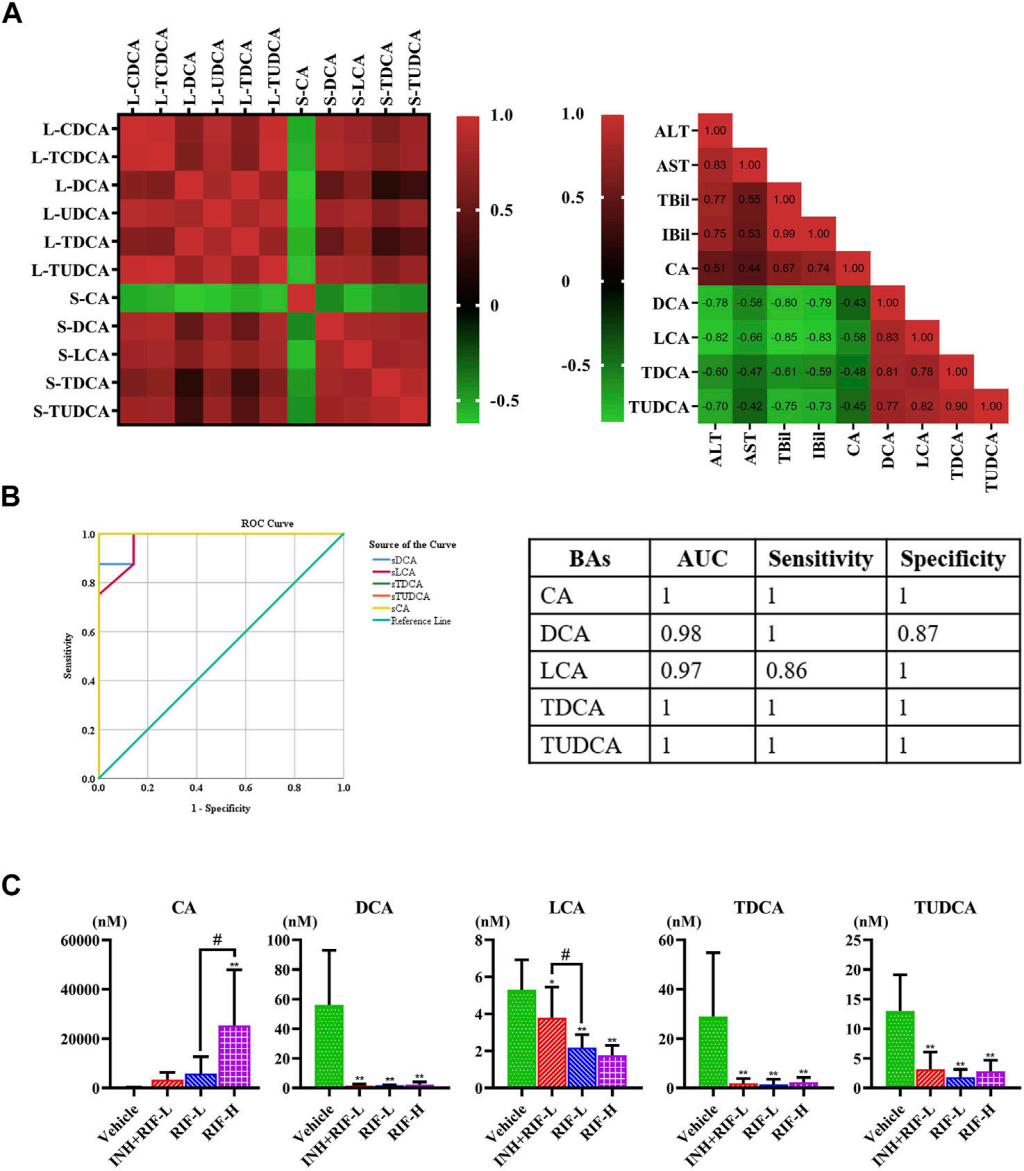
Screening targeted serum BAs **(A)** Correlations between serum and liver BAs (VIP ≥1) and between serum BAs and serum biochemical parameters, were analyzed using Spearman’s correlation analysis (*n* = 5–8) **(B)** ROC curve for serum CA, DCA, LCA, TDCA, and TUDCA **(C)** Comparative analysis of alterations in the serum BA levels in mice. Data are expressed as mean ± SD (*n* = 5–8). *p < 0.05, **p < 0.01 versus the vehicle group; #*p* < 0.05, ##*p* < 0.01 versus the RFP-L group.

Furthermore, the secondary BAs (DCA, LCA, TDCA, and TUDCA) were positively correlated with hepatic BAs (VIP ≥1), such as CDCA, TCDCA, DCA, UDCA, TDCA, and TUDCA. The primary BA (CA) was inversely associated with hepatic BAs (VIP ≥1). Pearson correlation analyses of BAs (CA, DCA, LCA, TDCA, and TUDCA) and the levels of ALT, AST, TBil, and IBil were conducted. CA showed a significant positive correlation with the levels of ALT, AST, TBil, and IBil (the correlation coefficients were 0.51, 0.44, 0.67, and 0.74, respectively). Levels of DCA, LCA, TDCA, and TUDCA were inversely associated with those of ALT, AST, TBil, and IBil, especially DCA and LCA. Moreover, the ROC curve analysis was performed for serum DCA, LCA, TDCA, TUDCA, and CA levels to distinguish abnormal liver injury from normal healthy cases without any injury **(** Figure 5 **)**. The cut-off value for sensitivity and specificity were determined among the different BAs in the ROC curve analysis. Our findings suggest that all of the above BAs are good indicators of RIF-induced liver injury with high sensitivity and specificity.

### 3.4 Effects of RIF on Fxr, Mrps, and UGT1a1 Expression

**FIGURE 6:**
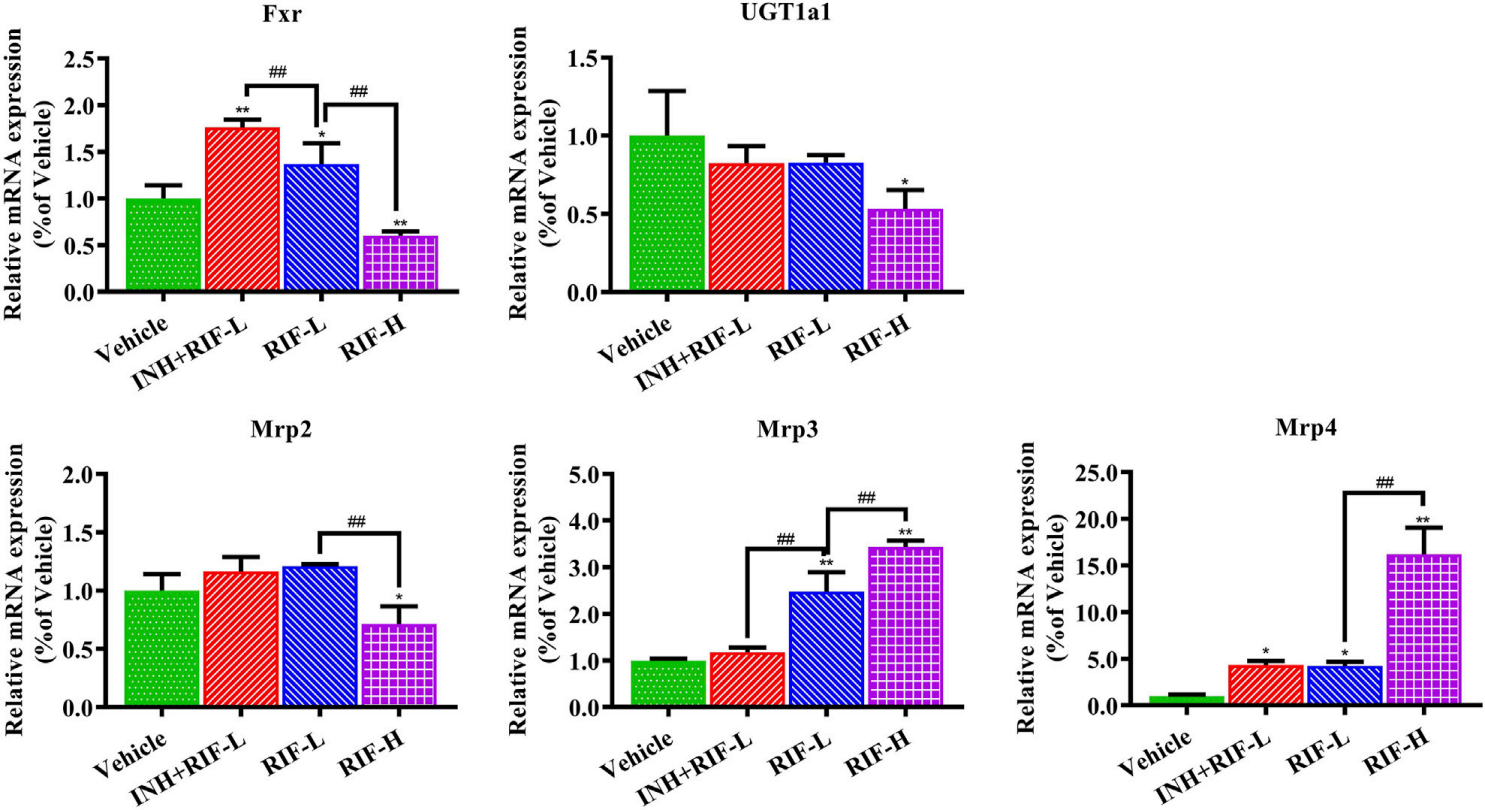
Changes in liver metabolic enzyme (UGT1a1) and Fxr-Mrps levels. Data are expressed as mean ± SD (*n* = 3). *p < 0.05, **p < 0.01 versus the vehicle group; #*p* < 0.05, ##*p* < 0.01 versus the RIF-L group.

Relative to the vehicle group, Fxr and Mrp4 messenger ribonucleic acid (mRNA) expressions increased in the INH + RIF-L and RIF-L groups, whereas that of Mrp3 was elevated in the RIF-L group. Fxr, Mrp2, and UGT1a1 mRNA expression levels notably decreased, whereas those of Mrp3 and Mrp4 increased in the RIF-H group in a dose-dependent manner (Figure 6).

## 4 Discussion

DILI is the primary reason for poor drug approval rates and drug withdrawal; it is also the most common severe adverse reaction to anti-TB therapy (Yang et al., 2020a). In a 14-days study on RIF dosing in mice, accumulation of lipids due to upregulation of peroxisome proliferator-activated receptor-γ was found to be the primary cause of RIF-induced toxicity at 177 mg/kg (LD_10_, equivalent to approximately 20 mg/kg in humans), while 442.5 mg/kg RIF caused another type of unspecified liver damage (LD_25_, equivalent to approximately 50 mg/kg in humans) (Kim et al., 2017). Thus, it is necessary to further investigate the pathogenesis at the early stages of liver injury caused by RIF at 442.5 mg/kg. On the other hand, previous studies indicated that the long-term administration of low-dose RIF in combination with INH was more likely to aggravate the occurrence of drug-induced liver injury than RIF alone (Devarbhavi et al., 2021). The underlying mechanisms of DILI caused by RIF or INH + RIF *via* LC-MS/MS-based targeted metabolomics approach had not been previously reported. As shown in Figure 2, our findings suggested that RIF aggravated BA metabolism disorders, thereby causing liver injury. The leading cause was intrahepatic cholestasis for RIF-L and INH + RIF-L, but extrahepatic cholestasis for RIF-H. Moreover, targeted metabolomic analysis of BAs and screening of biomarkers for liver injury showed that transportation of BAs, mediated by Fxr-Mrps, was closely related to RIF-induced liver injury.

Hepatic cholesterol is converted into CA and CDCA in classical and alternative metabolic pathways and then amidated with taurine or glycine. The conjugation with taurine and glycine lowers pKa and increases solubility to facilitate easier efflux into bile (Fiorucci et al., 2021). Monoanionic bile salts are secreted into the bile duct by the bile salt export pump, and dianionic bile salts; phase II-conjugated BAs are secreted into bile by Mrp2 (Meier and Stieger, 2002; Morgan et al., 2013). Under normal conditions, Mrp3 and Mrp4 are expressed in the basolateral membranes (Köck et al., 2014; Song et al., 2016). Mrp3 mediates the efflux of BAs, except for glutathione-conjugated BAs, from the basolateral membrane into the blood, while Mrp4 transports different types of BAs into the blood and plays a more critical role than Mrp3 (Jetter and Kullak-Ublick, 2020). Additionally, when primary BAs enter the terminal ileum through the bile duct, 95% of intestinal BAs are reabsorbed and transported back to the liver *via* the hepatic portal vein. Conjugated BAs are deamidated into free BAs in the intestine by bile salt hydrolase, expressed by *Bacteroides, Lactobacillus, Bifidobacterium,* and *Clostridium*. This is followed by transformation into secondary BAs (DCA, LCA, or UDCA) with 7α dehydrogenase, from *Clostridium* and *Eubacteria*, acting as the catalyst (Fiorucci et al., 2021).

Intrahepatic cholestasis might be observed in the RIF-L and INH + RIF-L groups, with significant elevation in the levels of primary BAs, including CA, CDCA, TCA, and TCDCA (Figure 3). Consistent with these results, Kim et al. (Kim et al., 2017) reported that RIF-induced hepatotoxicity did not significantly increase ALP level, indicating a type of mixed liver injury *via* cholestasis and hepatocellular injury. In our study, RIF-induced liver injury was evaluated by measuring ALT, AST, bilirubin changes and H&E staining according to relevant studies (Lin et al., 2019; Fan et al., 2019; Yang et al., 2020c; Yan et al., 2021). We did not measure or quantify CK19 + bile duct mass or ALP levels to analyze hepatotoxicity caused by biliary duct dilatation, which is a limitation in the present study. As shown in Figure 2, the hepatic cord and central vein arrangements were similar in the vehicle, RIF-L, and INH + RIF-L groups, except for slight nuclear disappearance and partial vacuoles. The degree of liver tissue injury in the INH + RIF-L group was slightly higher than that in the RIF-L group; however, the ALT and AST levels showed no significant changes. We speculated that these changes in BAs could reflect the early responses to liver injury better than the changes in liver enzymes, consistent with the results of a previous study (Slopianka et al., 2017). In addition, mild liver injury in the INH + RIF-L and RIF-L groups could be attributed to activation of the Fxr signaling pathway, which causes elevation of CA, CDCA, TCA, and TCDCA levels. The upregulation of Mrp4 expression in the INH + RIF-L group facilitated the BA efflux into the blood, whereas increased expression of Mrp3 and Mrp4 in the RIF-L group synergistically promoted the transport of BAs, resulting in lesser damage to the liver relative to the INH + RIF-L group.

Previous studies report that RIF inhibits the expression of UGT1a1 and the UGT1a1-related phase II metabolic pathway, which reduces the excretion of toxic components of RIF through Mrp2, and increases the risk of DILI (Cao et al., 2017). Additionally, our results showed that Fxr and Mrps mRNA expressions were significantly changed, a possible reason why the reduction in BA levels inhibited Fxr mRNA expression, in turn reducing those of Mrp2 and UGT1a1 and inducing a significant compensatory increase in Mrp3 and Mrp4 mRNA expression (Xu et al., 2005; Vanwijngaerden et al., 2011; Carino et al., 2020; Jetter and Kullak-Ublick, 2020). CA is a hydrophobic BA that induces hepatocyte death and cholestatic liver injury in mice (Wei et al., 2020). Previous studies demonstrate that the CA levels in patients with end-stage chronic cholestatic liver injury are substantially elevated (Fischer et al., 1996; Fu et al., 2022). Slopianka M et al. suggest that serum CA, CDCA, DCA, beta muricholic acid, and UDCA levels may serve as new DILI biomarkers (Slopianka et al., 2017). It is also believed that serum CA, TCA, and GCA can be DILI biomarkers with higher specificity relative to standard biomarkers, such as ALT (Luo et al., 2014). On establishing the PLS-DA model of BAs, in the vehicle and RIF-H groups, we found significant decreases in the levels of serum biomarkers (DCA, LCA, TDCA, TUDCA) which were positively correlated with liver BAs (CDCA, TCDCA, DCA, UDCA, TDCA, and TUDCA). We speculated that the significant decreases in the levels of secondary BAs and the ratio of secondary to primary BA levels could be attributed to the considerable induction of *Bacteroides* and reduction in *Clostridium* and *Eubacter* abundance (Namasivayam et al., 2017; Khan et al., 2019). RIF administration suppressed the biotransformation of 7α dehydrogenase-mediated dehydroxylation and intestinal bile salt hydrolase production, thereby reducing the biotransformation of CA and CDCA into DCA and LCA, respectively (Fiorucci et al., 2021). Interestingly, significantly elevated serum primary BA (CA) levels negatively correlated with those of liver BAs. Consistent with previous research, the elevation of serum CA levels may be related to increased intrahepatic CA efflux into the blood *via* Mrp3 and Mrp4 (Matye et al., 2021). In addition, the reduced secondary BA generation led to lower levels of CA and other BAs in the RIF-H group relative to those in the vehicle group. Disruption of liver BA and blood BA homeostasis results in liver injury. ROC curves for serum CA, DCA, LCA, TDCA, and TUDCA were highly sensitive, specific, and strongly correlated with ALT and AST, suggesting a particular value indicating RIF-induced DILI. Our study provided further insights into the pathogenesis of RIF-induced liver injury and a theoretical basis for DILI prevention and rational clinical application in anti-TB therapy.

In addition, it has been reported that the activation of Fxr can inhibit the nuclear receptor nuclear factor-κB (NF-κB) signaling pathway, inhibiting the expression of inflammatory factors tumor necrosis factor-α, interleukin-1β, and interleukin-6, which play an anti-inflammatory role (Wang et al., 2008; Ming et al., 2021). Wang and others (Wang et al., 2008) also described negative crosstalk between the FXR and NF-κB signaling pathways in the hepatic inflammatory response. However, Yu et al. found that GW4064 can activate the Fxr signaling pathway and the NF-κB signaling pathway in normal human gastric epithelial cells (Yu et al., 2019). Consistent with these results, Lee et al. (Lee et al., 2011) reported that downregulation of FXR in pancreatic carcinoma cells decreases NF-κB activity and consequently inhibits its target genes, indicating that NF-κB may participate in the regulation of FXR activity (Gadaleta et al., 2011), involving a positive crosstalk mechanism. In summary, the mechanisms between Fxr regulation of BA metabolism, anti-inflammatory and NF-κB causing inflammatory damage are complex and remain unclear. Further researches are required to explore and verify in the future.

## 5 Conclusion

Exposure to different doses of RIF induces various types of liver injury. RIF-H treatment might lead to extrahepatic cholestasis, with significantly elevated serum CA levels. In this study, CA, DCA, LCA, TDCA, and TUDCA were identified as potential biomarkers for the early detection of RIF-induced liver injury, reflecting initial responses to and the severity of the liver injury. The hepatic toxicity mechanisms of RIF may be related to the liver metabolic enzymes (UGT1a1) and BA transporters, including Mrp2, Mrp3, and Mrp4.

## Data Availability

The original contributions presented in the study are included in the article/Supplementary Materials, further inquiries can be directed to the corresponding author.

## Glossary

ALT: alanine: aminotransferase
AST: aspartate aminotransferase
ALP: alkaline phosphatase
ALT: alanine: aminotransferase
mRNA: messenger ribonucleic acid
RNA: ribonucleic acid
RIF: rifampicin
BA: bile acid
Fxr: farnesoid x receptor
Mrp: multidrug resistance-associated protein
UGT: uridine diphosphate-glucuronosyltransferase
TB: Tuberculosis
DILI: drug-induced liver injury
CA: cholic acid
CDCA: chenodeoxycholic acid
DCA: deoxycholic acid
LCA: lithocholic acid
UDCA: ursodeoxycholic acid
HDCA: hyodeoxycholic acid
TCA: taurocholic acid
TCDCA: taurochenodeoxycholic acid
TDCA: taurodeoxycholic acid
TLCA: taurolithocholic acid
TUDCA: tauroursodeoxycholic acid
THDCA: taurohyodeoxycholic acid
GCA: glycocholic acid
GCDCA: glycochenodeoxycholic acid
GDCA: glycodeoxycholic acid
GUDCA: Glycoursodeoxycholic acid
TBil: total bilirubin
IBil: indirect bilirubin
LC-MS/MS: liquid chromatography-tandem mass spectrometry
IS: internal standard; PCR, polymerase chain reaction
PLS-DA: partial least squares discriminant analysis
VIP: variable importance value
ROC: receiver operating characteristic
NF-κB: nuclear factor-κB
