# Supplementary figures and images for: Screening of Biomarkers and Toxicity Mechanisms of Rifampicin-Induced Liver Injury Based on Targeted Bile Acid Metabolomics
Source: Front Pharmacol. 2022 Jun 10;13:925509. doi: 10.3389/fphar.2022.925509 (PMC9226894; doi:10.3389/fphar.2022.925509)

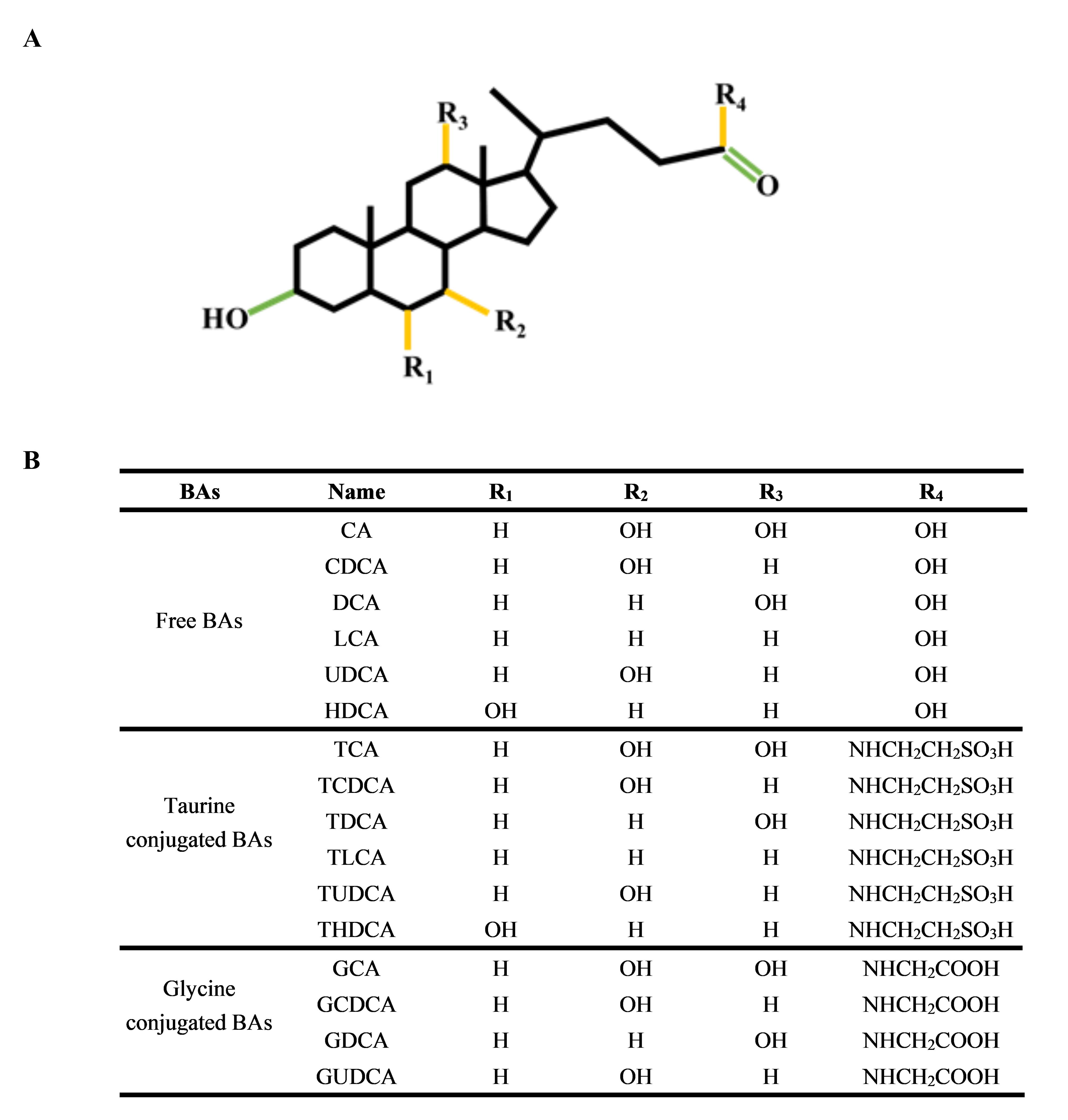

Supplement: Supplementary file 1 [file Image1.JPEG]
